# Influence of Gas Supply Changes on the Formation Process of Complex Mixed Gas Hydrates

**DOI:** 10.3390/molecules26103039

**Published:** 2021-05-19

**Authors:** Mengdi Pan, Judith M. Schicks

**Affiliations:** 1 GFZ German Research Centre for Geosciences, Telegrafenberg, 14473 Potsdam, Germany; mengdpan@gfz-potsdam.de; 2 Department for Earth and Environmental Sciences, University of Potsdam, 14476 Potsdam, Germany

**Keywords:** mixed gas hydrates, hydrate formation process, coexisting phases, in situ Raman spectroscopy, gas supply conditions

## Abstract

Natural gas hydrate occurrences contain predominantly methane; however, there are increasing reports of complex mixed gas hydrates and coexisting hydrate phases. Changes in the feed gas composition due to the preferred incorporation of certain components into the hydrate phase and an inadequate gas supply is often assumed to be the cause of coexisting hydrate phases. This could also be the case for the gas hydrate system in Qilian Mountain permafrost (QMP), which is mainly controlled by pores and fractures with complex gas compositions. This study is dedicated to the experimental investigations on the formation process of mixed gas hydrates based on the reservoir conditions in QMP. Hydrates were synthesized from water and a gas mixture under different gas supply conditions to study the effects on the hydrate formation process. In situ Raman spectroscopic measurements and microscopic observations were applied to record changes in both gas and hydrate phase over the whole formation process. The results demonstrated the effects of gas flow on the composition of the resulting hydrate phase, indicating a competitive enclathration of guest molecules into the hydrate lattice depending on their properties. Another observation was that despite significant changes in the gas composition, no coexisting hydrate phases were formed.

## 1. Introduction

Natural gas hydrates are ice-like crystalline solids composed of a three-dimensional network of water molecules. The water molecules form well-defined cavities via hydrogen bonds that are stabilized by the inclusion of guest molecules [1,2]. The formation of gas hydrates in nature requires low temperatures, elevated pressures, and substantial amounts of gases and water, which limits their global distribution to marine sediments at all active and passive continental margins, permafrost areas, and deep lakes [3,4]. Although methane (CH_4_) is in general the predominant gas molecule in hydrate cavities, other natural gas compounds such as C_2_–C_5_, carbon dioxide (CO_2_), and hydrogen sulfide (H_2_S) can also be enclathrated into the water cavities. Depending on the size of the guest molecule, three main types of crystal hydrate structures could be detected in nature: the cubic structures I (sI) and II (sII) and the hexagonal structure H (sH) [2]. Small guest molecules such as CH_4_ and CO_2_ form sI hydrates, whereas larger molecules such as propane (C_3_H_8_) or iso-butane (iso-C_4_H_10_) form sII hydrates. Even larger guests such as iso-pentane or neo-hexane can only enter the large cavities of sH hydrates in the presence of a help gas such as CH_4_. 

In natural environments, gas hydrates usually contain a mixture of the above-mentioned gases, which originate from microbial activities and/or thermogenic conversion of organic matters from deeper sediments. The formation of gas hydrates from a gas mixture, however, may result in a coexistence of hydrate phases with different structures and compositions. This has already been verified in natural gas hydrate deposits. In 2007, a complex hydrate sample containing n-pentane and n-hexane in addition to CH_4_ and lighter hydrocarbons were identified from Barkley Canyon on the northern Cascadian margin. For the first time the coexistence of sII and sH hydrate phases could be proved in nature [5]. Later, in 2010, Klapp et al. reported the occurrence of sI and sII gas hydrates as coexisting phases from the Chapopote Knoll in the southern Gulf of Mexico [6]. Similar results were observed from the South China Sea by Wei et al. [7], who analyzed gas hydrate samples and confirmed the coexistence of sI and sII gas hydrates. Even hydrate samples from Lake Baikal showed the coexisting hydrate phases with different structures and compositions [8]. Under certain conditions the formation of a more or less homogeneous mixed hydrate phase may be difficult in the presence of a gas mixture, as indicated by the above-mentioned examples. However, the reason for the formation of coexisting hydrate phases can be diverse and are not yet fully clarified. 

The occurrence of mixed gas hydrates was also demonstrated in Qilian Mountain permafrost (QMP), in China, in 2008. Gas hydrates were discovered in the global mid-latitude permafrost region of high elevation. A special feature of these hydrate deposits is their occurrence at a shallow depth below thin permafrost. In addition, it is characterized by more complicated gas components compared to other well-documented permafrost hydrate reservoirs [9,10,11]. The composition and carbon isotopes of gases released from gas hydrates suggest that the feed gas source for the hydrate reservoir is composed mainly of deep or crude oil-concomitant gases originating from the coal layers of the Middle Jurassic period, occasionally mixed with microbial sources in shallower depths [12,13,14,15]. Fracture-filling gas hydrates are the main reservoir type and manifest as thin layers, flakes, or blocks on the fractured surface of siltstone, mudstone, and oil shales. Previous studies on the geological settings of the Qilian gas hydrate system unveiled a complex system of faults over a deep potential hydrocarbon reservoir [16,17]. The hydrocarbon reservoir is therefore mainly controlled by faults and fractures with discontinuous distributions in a vertical direction [16,18,19]. The characteristic of hydrate distribution in the lateral areas between drilling holes is also not apparent due to the rock fracture system. In this case, gas mixtures derived from deep hydrocarbon reservoirs are being transported from the place of origin at greater depths through fractures to the shallower hydrate stability zone for the formation of gas hydrates. 

Research has been carried out to investigate the hydrate samples recovered from the drilling zone of Qilian Mountain permafrost. It turns out that the gases obtained from hydrate dissociation contain about 60% CH_4_. In addition to CH_4_, there is a high percentage of other gas components such as C_2_H_6_, C_3_H_8_, C_4_H_10_ and even heavier hydrocarbons [11]. These larger molecules occupy large cavities of the hydrate structures, whereas CH_4_ molecules are mainly in small cavities, as indicated by the Raman spectroscopic measurements, suggesting that the gas hydrates belongs to structure II hydrates [18,19]. The encasement of higher hydrocarbons besides CH_4_ into the hydrate structures results in a shift in the stability conditions to higher temperatures and lower pressures, and an increase in the dissociation enthalpy compared to pure CH_4_ hydrates [20,21,22]. Thus, the occurrence of higher hydrocarbons in the feed gas phase is a prerequisite for the formation of a hydrate phase under these mild pressure and temperature conditions. However, the feed gas mixtures are highly dependent on the local conditions of the hydrate system. It is likely that both the changes in the gas migration pathways and hydrocarbon reservoirs will impede the upward gas flow or vary the feed gas composition. The formation of the mixed gas hydrates from a changing gas phase may lead to the coexistence of different gas hydrate phases. Regarding hydrate formation in nature, it is questionable whether the feed gas phase is available continuously or whether it is disrupted. The limitation of gas migration may be caused by the formation of hydrate plugs in pore necks or fractures, permeability changes due to mineral diagenesis, or periodical geotectonic movements in geological times. Against such background, the gas supply condition plays a significant role in the formation process of mixed gas hydrates.

In this study, we present the results from our experiments simulating three potential different hydrate formation conditions based on the background in QMP, an open system with a continuous gas flow, a semi-closed system with an interrupted outlet gas flow, and a closed system with no further gas supply after initial pressurization. In situ Raman spectroscopic measurements and microscopic observations were applied for the investigations into the mixed gas hydrates formed from a gas mixture containing CH_4_, C_2_H_6_, C_3_H_8_, iso-C_4_H_10_, and n-C_4_H_10_ throughout the three parallel tests. The results revealed the effects of different gas supply conditions on the composition of the resulting hydrate phase. However, the coexistence of different hydrate phases was not observed with a changing gas phase, at least not during the experimental period. This study provides important information about the gas enclathration into hydrate structures on a µm scale, which may enhance our understanding of the complicated mixed hydrate formation process.

## 2. Results

### 2.1. Raman Spectra of Mixed Gas Hydrates

**Figure 1:**
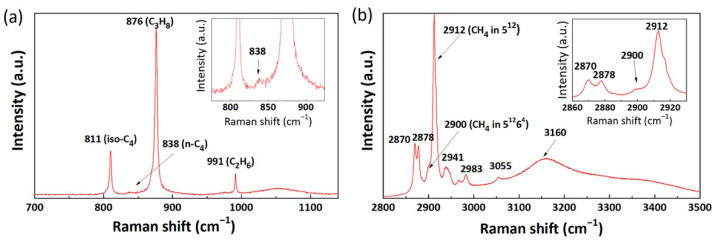
Raman spectra of the mixed gas hydrates. (**a**) C−C stretching vibrational modes ranged from 700–1350 cm^−1^, and (**b**) C−H stretching vibrational modes and O−H stretching modes ranged from 2800–3500 cm^−1^. The inset in Figure 1a shows the enlarged Raman band at 838 cm^−1^, whereas the inset in Figure 1b depicts the enlarged Raman bands ranging from 2860–2940 cm^−1^.

Experimental investigations were performed by applying in situ Raman spectroscopy at 274 K and 2.20 MPa for the gas mixture containing CH_4_, C_2_H_6_, C_3_H_8_, iso-C_4_H_10_, and n-C_4_H_10_. Single-point Raman measurements on more than 12 crystals showed the formation of sII mixed hydrates with the incorporation of CH_4_ mainly in small 5^12^ cavities and other hydrocarbon molecules in large 5^12^6^4^ cavities. Figure 1 depicts the Raman spectra of the mixed gas hydrates in the region between 700 cm^−1^ and 1100 cm^−1^ (related to C−C stretching vibrations, Figure 1a), between 2800 cm^−1^ and 3500 cm^−1^ (related to C−H stretching and O−H stretching vibrations, Figure 1b). The measured and assigned Raman band positions and corresponding literature data are summarized in Table 1.

Between 700 cm^−1^ and 1100 cm^−1^, four characteristic Raman bands were recorded, and the largest Raman band, at 876 cm^−1^, could be assigned as the C−C stretching vibration of C_3_H_8_ enclathrated into the large cavities of the sII hydrate [23]. The Raman band with the second highest intensity was detected at approximately 991 cm^−1^. Notably, the Raman band that arose at 1000 cm^−1^ was reported by previous researches to be an indication of C_2_H_6_ encased in the large cavities of the sI hydrate, whereas the Raman band at 991 cm^−1^ was for C_2_H_6_ in the sII hydrate [24,25]. Hence, we assigned the obtained 991 cm^−1^ Raman band to the C_2_H_6_ encased in the large 5^12^6^4^ cavities of the sII hydrate. The positions of these two Raman bands agreed very well with the data reported by Lu et al., who also performed Raman spectroscopic measurements on natural gas hydrate samples recovered from QMP [10]. The striking Raman band in the C−C area for iso-C_4_H_10_ was located at around 811 cm^−1^. It was consistent with the Raman data for iso-C_4_H_10_ encased in the hydrate phase reported by Klapp et al. [6], Subramanian and Sloan [26], and Uchida et al. [27]. The intensity of the Raman band at 838 cm^−1^ was extremely low and thus zoomed in, as shown in the inset of the figure. This Raman band was related to the n-C_4_H_10_ in the large 5^12^6^4^ cavities of the sII hydrate phase [6]. The relatively low signal-to-noise ratio can be attributed to the low concentration of n-C_4_H_10_ in the hydrate phase.

The existence of the CH_4_ molecules in both cavities of the sII hydrate was exhibited in the Raman spectrum of the C−H stretching vibration mode. In Figure 1b, a prominent Raman band can be observed at 2012 cm^−1^ for the CH_4_ in 5^12^ cavities. The small shoulder at approximately 2900 cm^−1^ may be an indication of a small amount of CH_4_ molecules in 5^12^6^4^ cavities of the sII hydrate. A minor Raman band at around 3055 cm^−1^ (Figure 1b), indicative of CH_4_ in the hydrate phase [28], could also be detected. It is important to mention that the Raman bands of CH_4_ in this complex sII mixed hydrate system appeared to have a slight redshift as compared to previous sII hydrate systems with fewer components [21,27,29]. However, it was in good agreement with previous Raman observations on natural hydrate samples collected from the QMP hydrate reservoir [18]. The band position difference could be attributed to the influence of higher hydrocarbon molecules in adjacent cavities. The double Raman bands that appeared at around 2870 cm^−1^ and 2878 cm^−1^ were assigned to the C−H stretching mode of the C_3_H_8_ molecules in sII hydrates. They were good indicators of C_3_H_8_ in hydrate lattices [23]. Further signals at 2941 cm^−1^, 2967 cm^−1^, and 2983 cm^−1^ were probably those of the C−H stretching vibrations of C_2_H_6_, iso-C_4_H_10_, and n-C_4_H_10_ [6]. Additionally, Figure 1b shows the O-H stretching vibration for water molecules (3000–3500 cm^−1^), which could be analyzed to further verify the formation of a hydrate phase. A broad Raman band was observed at around 3160 cm^−1^, which corresponded to increased hydrogen bonds due to the formation of a well-structured hydrogen-bonded network in the hydrate phase [30]. In summary, these results confirm that sII hydrates were formed from the gas mixture under the chosen experimental conditions. C_2_H_6_, C_3_H_8_, iso-C_4_H_10_, and n-C_4_H_10_ molecules were encaged only in the large cavities (5^12^6^4^) of the sII hydrate, whereas CH_4_ predominantly occupied the small 5^12^ cavities of the sII hydrate.

### 2.2. Enclathration Sequence of Gas Components into the Hydrate Structures

**Figure 2:**
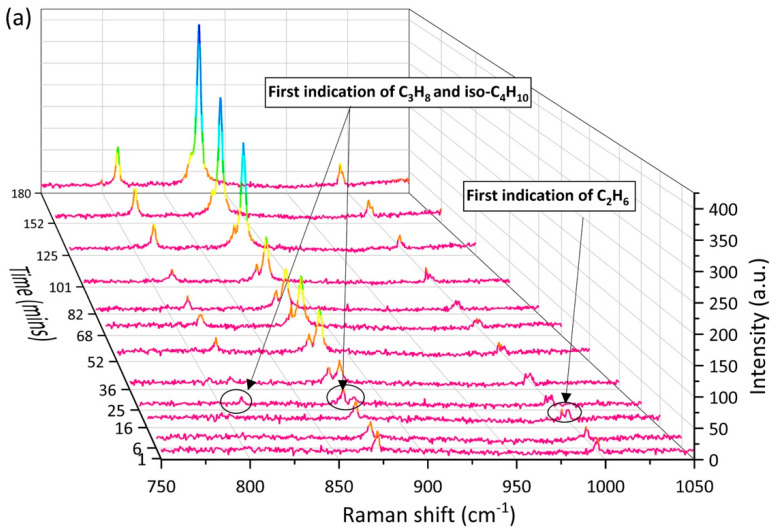

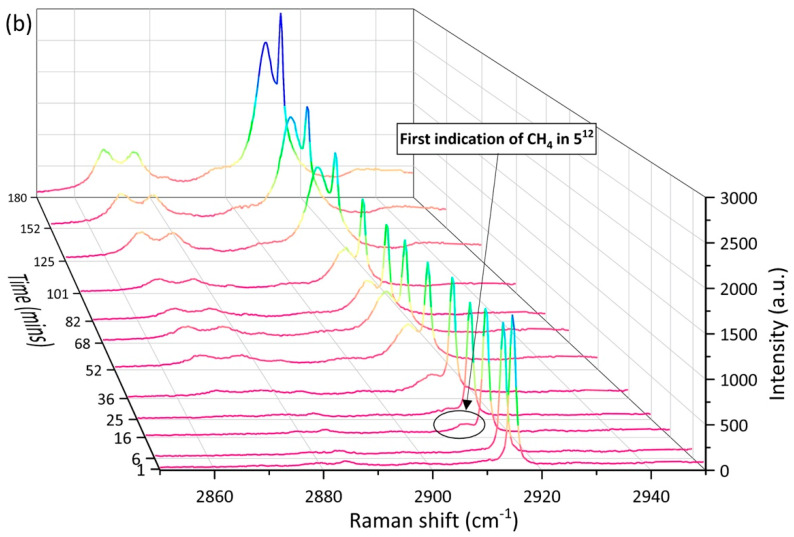
Time-resolved overview of Raman spectra in the (**a**) C–C stretching vibrational modes ranged from 700–1100 cm^−1^, and (**b**) C–H stretching vibrational modes ranged from 2840–2950 cm^−1^, as recorded on the surface of a hydrate crystal in the open system during the first 180 min.

Real-time Raman spectra provided information on the hydrate phase during the experiments and clarified the enclathration sequence for different components being encased into the hydrate cavities. It is worth mentioning that the sequence refers to the order of Raman bands being detected for each specific component by the Raman system and observed in the Raman spectra. Even though the induction time that indicated the first detectable signal for the enclathration of gas molecules into a hydrate cavity varied significantly under different gas supply conditions, the enclathration sequence was generally similar and independent of the gas supply conditions. Taking the results from one test as an example, Figure 2 shows the compiled Raman spectra taken from the surface of a single hydrate crystal in the open system. The crystal was closely monitored during the first 3 h. At the beginning of the experiment, only Raman bands at 2917 cm^−1^, 993 cm^−1^, and 869 cm^−1^ were detected in the system which could be assigned to CH_4_, C_2_H_6_, and C_3_H_8_ in the gas phase, respectively. It should be noted that with the use of a confocal system, Raman signals for the surrounding gas phase were limited, however, not completely avoided during the measurements of hydrate phase, especially when the measurements were carried out on the surface of the crystal.

The first indication of C_2_H_6_ encased in the large 5^12^6^4^ cavities of the sII hydrate came at around 16 min, when a tiny Raman band was observed at 991 cm^−1^ next to the Raman band at 993 cm^−1^ assigned to C_2_H_6_ in the gas phase. At the same time, a shoulder at 2912 cm^−1^ could be observed in the Raman spectra, suggesting the enclathration of CH_4_ molecules into the small 5^12^ cavities of the sII hydrate. After around 25 min, two small Raman bands occurred at 811 cm^−1^ and 876 cm^−1^, indicating the formation of a mixed gas hydrate phase encasing iso-C_4_H_10_ and C_3_H_8_ in the large 5^12^6^4^ cavities of the sII hydrate. Unfortunately, we were not able to detect the Raman band for n-C_4_H_10_ due to its low concentration in both the gas phase and hydrate phase during the measuring time period. With an increasing number of guest molecules being trapped in the hydrate cavities over time, the intensities of Raman bands also increased as the experiment proceeded.

### 2.3. Composition of the Feed Gas Mixture

It should be noted that the defined composition of the gas mixture used in the experiments (±0.1 mol% rel) was only guaranteed by the manufacturer Rießner-Gase GmbH for storage at certain temperatures and for a limited period of time. Since it was therefore possible that the composition of the gas phase had changed over time during transportation, storage, or during the experiments and their repetitions, Raman spectroscopic measurements were carried out on the pure gas phase without water (274 K, 2.20 MPa) before the experiments were conducted to determine the actual composition of the gas mixture flowing into the pressure cell. The composition of the gas phase at the beginning of the experiments and the changes of the gas phase composition during the experiments are presented in the following paragraphs. Once the mixed hydrates started to form from the feed gas mixture, the inlet and outlet valves located outside the pressure cell were set to the desired position (either open or closed) to control the supply gas flow. These changes to the gas flow were maintained throughout the whole formation process. In all three experimental systems, Raman spectra continuously recorded potential changes in composition in the gas phase over the formation period.

In the open system, investigations into the gas phase showed no significant changes over the whole formation period, indicating a stable gas flow. The absolute pressure in the sample chamber was maintained at 2.20 MPa. In addition, the composition of the feed gas was the same as the initial pure gas, which was composed of 71.2 ± 2.2 mol% CH_4_, 11.3 ± 0.9 mol% C_2_H_6_, 15.4 ± 1.5 mol% C_3_H_8_, 1.5 ± 0.3 mol% iso-C_4_H_10_, and 0.6 ± 0.2 mol% n-C_4_H_10_.

**Figure 3:**
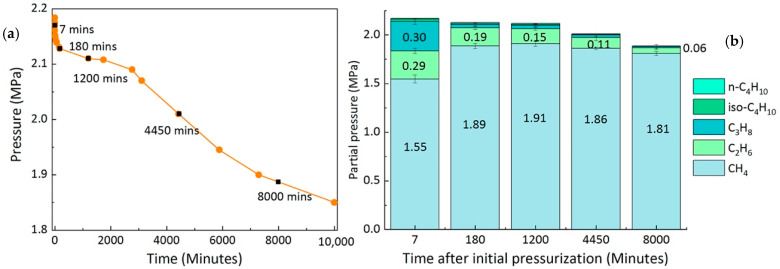
Changes in the absolute pressure of the sample cell (**a**) and the partial pressure changes of each component measured at different times in the closed system (**b**). The black squares in Figure 3a indicate the specific measuring time during the ongoing experiments.

In comparison, the ongoing formation process in the completely closed system after initial pressurization resulted in a varying gas phase in the sample cell. Figure 3a depicts the changes in absolute pressure in the closed system once the formation process started. Initially, the pressure in the sample cell was 2.20 MPa. With the enclathration of guest molecules into the hydrate cavities, the absolute pressure decreased to 2.13 MPa within the first 3 h. Thereafter, it became steady at around 2.11 MPa, a pressure that was higher than the equilibrium pressure of the mixed gas hydrates at 274 K. This intermediate plateau lasted for several hours before the hydrate continued to form slowly until it reached a steady state. Here, we calculated the partial pressure of each gas component from five Raman measurements taken at different times during the ongoing process (7 min, 180 min, 1200 min, 4450 min, and 8000 min after initial pressurization), as shown in Figure 3b. Within the first 180 min, the composition of the gas phase changed significantly and became more CH_4_-rich, since C_3_H_8_ molecules were preferentially encased in the hydrate phase and therefore removed from the vapor phase. This resulted in a rapid decrease in pressure during the first seven minutes, as shown in Figure 3a. After around 180 min, the formation process slowed down, as indicated by the “plateau” period. Thereafter, hydrocarbons (C_2_H_6_, C_3_H_8_, iso-C_4_H_10_, and n-C_4_H_10_) were further removed from the vapor phase, but at a slower rate compared to that within the first 180 min. Finally, the gas phase was composed of more than 90 mol% CH_4._ This observation can be summarized as a two-step formation process, which was consistent with previous findings by Uchida et al. [23] on the CH_4_-C_3_H_8_ mixed gas hydrates formed in a batch-type reactor.

As for the semi-closed system where the outlet gas was interrupted, the gas phase change was less pronounced than that in the closed system due to the supplemented gas flow from the inlet. However, it was also slightly different from the gas in the open system, containing a higher concentration of C_2_H_6_ and C_3_H_8_ but a lower concentration of CH_4_ from the beginning of the experiment. The absolute pressure decreased due to the enclathration of gas molecules into hydrate structures, likely accompanied by a depletion of certain gas components in the gas phase. With a pressure decrease of more than 0.05 MPa, the pressure regulator was activated and the pressure in the sample cell was corrected to 2.20 MPa. However, the depletion of certain gas components between two pressure regulation surges was not detected with the individual measurements. The average gas composition was around 67.0 ± 1.8 mol% CH_4_, 13.0 ± 1.3 mol% C_2_H_6_, 17.6 ± 2.0 mol% C_3_H_8_, 1.6 ± 0.3 mol% iso-C_4_H_10_, and 0.8 ± 0.5 mol% n-C_4_H_10_ during the ongoing process.

### 2.4. Composition of the Mixed Gas Hydrates

Our systematic Raman spectroscopic investigations on the mixed gas hydrates revealed the whole formation process of the sII hydrate by detecting the enclathration of CH_4_ into small 5^12^ cavities and C_2_H_6_, C_3_H_8_, iso-C_4_H_10_, and n-C_4_H_10_ in large 5^12^6^4^ cavities. In this chapter, we determined the composition of the formed mixed gas hydrates under different formation conditions with regard to the gas supply. It is noteworthy that the accuracy of the Raman measurements was calibrated before the experiment on a mixed gas hydrate crystal with a few measuring points. A good reproducibility of the measurements was confirmed, with an average standard deviation of 0.2 mol% for the measuring points over three repeated tests. Therefore, the scattered composition of hydrate crystals detected during the experiments indicated the heterogeneity of the hydrate phase in the systems.

**Figure 4:**
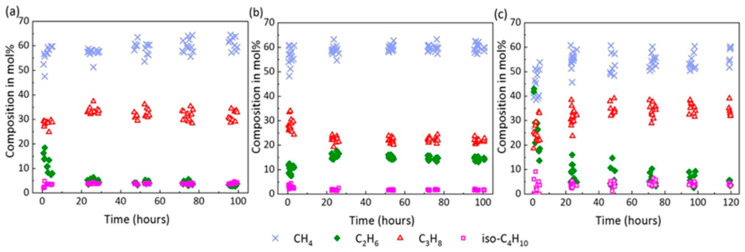
Composition changes measured on the surface of selected mixed hydrate crystals over the formation process in the open system (**a**), the closed system (**b**), and the semi-closed system (**c**).

**Figure 5:**
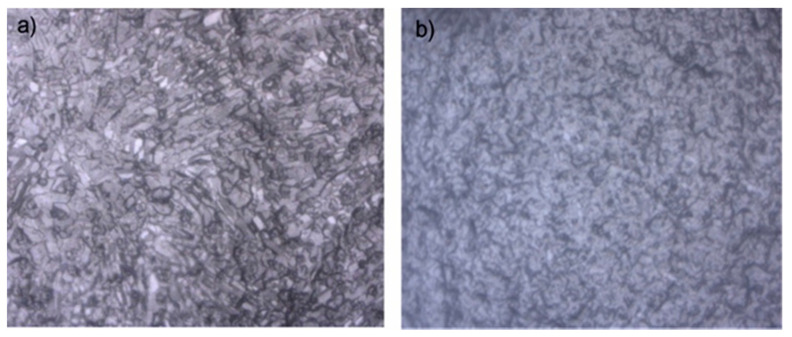
Microscopic pictures of hydrates crystals formed (**a**) in the open system and (**b**) in the closed system at *t* = 30 min after initial pressurization.

Figure 4 shows the changes in composition in the hydrate phase based on single-point measurements on the surfaces of 12 selected hydrate crystals in three systems. Surprisingly, the concentration of C_2_H_6_ in the hydrate phase was higher at the very beginning of the hydrate formation process but decreased over time, whereas the content of CH_4_ in the hydrate phase slightly increased with time in the open system (Figure 4a). As for C_3_H_8_ and iso-C_4_H_10_, the concentrations did not change significantly over time. Unfortunately, Raman bands for the n-C_4_H_10_ in the hydrate phase were either not detectable or had low intensities, and were therefore excluded from this figure. In the closed system that was carried out under identical p-T conditions but with no gas supply after initial pressurization, the formation process slowed down, as indicated by the microscopic observations. Figure 5 shows two microscopic pictures of hydrate crystals taken from the open (Figure 4a) and closed system (Figure 4b) after 30 min. Instead of transforming to well-developed sharp-faceted euhedral crystals, the crystals in the closed system were small and without sharp edges after the same time. Apparently, the concentration of C_2_H_6_ and CH_4_ in the closed system increased on the first day, whereas the concentration of C_3_H_8_ and iso-C_4_H_10_ declined, as seen in Figure 4b. Only a small variation was observed for the 12 hydrate crystals regarding the composition at a steady state. In the semi-closed system (Figure 4c), the composition for the selected hydrate crystals scattered over a wide range, especially on the first two days, showing a high heterogeneity of the hydrate phase. It turned out that the concentration of C_2_H_6_ declined sharply on the first day of the experiment, whereas the concentration of CH_4_ increased. This trend was consistent with the findings in the open system (Figure 4a). Afterwards, the composition of the hydrate phase stabilized and the whole system reached a steady state. The data presented in Figure 4 were obtained from one of the tests for each gas supply condition. However, a similar phenomenon was observed when repeating the experiments, as can be seen in the supporting information (Figure S1).

As demonstrated from the above Figure 4, significant changes in the concentration of guest molecules always occurred within the first day of the experiment. After 5–6 days, the systems were assumed to reach steady states when no significant changes regarding the composition of the hydrate phase were observed. Therefore, the average composition of the hydrate phase was analyzed after the first 30 min, after the first 4 h, and after 5 days at a steady state. The results are summarized in Table 2 and presented in Figure 5. Errors bars were calculated from the standard deviation of average compositions over the repeated experiments.

**Figure 6:**
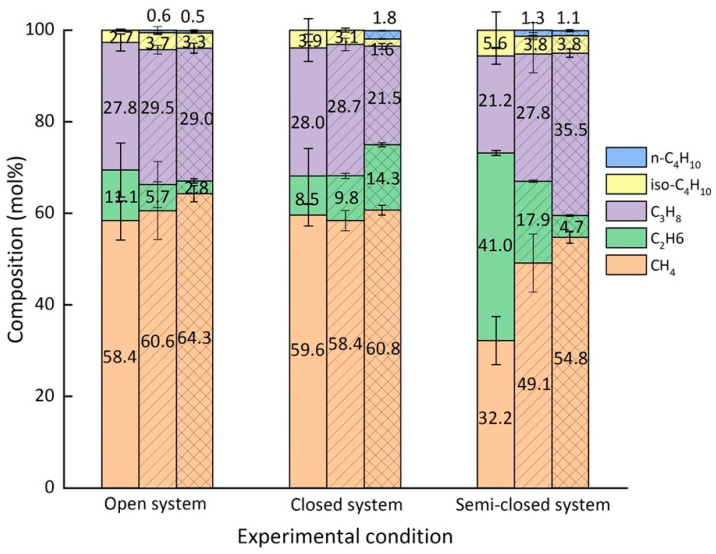
Average composition of the measured hydrate crystals formed under different gas supply conditions after 30 min (blank), after 4 h (shaded slash), and after 5 days (oblique line grid). The standard deviations of the average composition in repeated experiments were used for the descriptive error bars.

In the open system, the most noticeable change with regard to the composition of the hydrate phase after 30 min and 4 h was the decrease in the proportion of C_2_H_6_ (Figure 6). It should be noted, however, that the composition of the crystals varied much more after 30 min than after 4 h. As seen from the left side of Figure 6, larger molecules such as C_3_H_8_ and iso-C_4_H_10_ were preferentially enriched into the hydrate phase from the beginning of hydrate formation compared to the corresponding concentrations of these components in the gas phase. Finally, the average composition of the resulting mixed hydrate crystal at a steady state was around 64.3 mol% CH_4_, 2.8 mol% C_2_H_6_, 29.0 mol% C_3_H_8_, 3.3 mol% iso-C_4_H_10_, and 0.5 mol% n-C_4_H_10_ (Figure 6). This composition was very close to the equilibrium composition of the hydrate phase calculated with CSMGem, which was 61.6 mol% CH_4_, 2.6 mol% C_2_H_6_, 30.5 mol% C_3_H_8_, 5.0 mol% iso-C_4_H_10_, and 0.5 mol% n-C_4_H_10_. CSMGem is a software developed by the Colorado School of Mines and is widely used for the prediction of thermodynamically stable hydrate structures and cage occupancies in specific systems [2]. Our experimental results clearly indicate a preferred enclathration of the C_2_H_6_ into the hydrate cavities at the beginning of the experiment. However, the ability of C_2_H_6_ molecules to stabilize the cavities was supposed to be less pronounced compared to C_3_H_8_ or iso-C_4_H_10_, which led to a decrease in the concentration at the end of the experiment, indicating that C_2_H_6_ was not enriched in the hydrate phase [31]. In contrast, C_3_H_8_ and iso-C_4_H_10_ were enriched in the hydrate phase; their concentration was higher in the hydrate phase than the feed gas phase. Apart from C_2_H_6_, the proportion of the other components in the hydrate phase increased slightly as the experiment proceeded.

In the closed system the composition of the hydrate crystals also varied significantly after the first 30 min. n-C_4_H_10_ could not be detected in the hydrate phase at that time. The formed hydrate phase had a similar composition as that formed in the open system. Thereafter, the composition of the hydrate phase slowly adapted to the changes in the gas phase, which was depleted in C_3_H_8_. The hydrate phase at a steady state contained around 60.8 mol% CH_4_, 14.3 mol% C_2_H_6_, 21.5 mol% C_3_H_8_, 1.6 mol% iso-C_4_H_10_, and 1.8 mol% n-C_4_H_10_. Obviously, more C_2_H_6_ was incorporated into the hydrate phase and n-C_4_H_10_, which, though only encased in the hydrate phase in small quantities, was detected in the hydrate phase at steady state (Figure 6). The results indicate that a limited gas supply led to the enclathration of the components that were not preferred in the open system (e.g., C_2_H_6_ and n-C_4_H_10_). Accordingly, the proportions of C_3_H_8_ and iso-C_4_H_10_ that were preferentially enriched in the hydrate phase in the open system were lower in the hydrate phase in the closed system.

Due to a frequent re-adjustment of the gas flow in the semi-closed system, an average composition of 32.2 mol% CH_4_, 41.0 mol% C_2_H_6_, 21.2 mol% C_3_H_8_, and 5.6 mol% iso-C_4_H_10_ was recorded in the hydrate phase after the first 30 min. C_2_H_6_ and iso-C_4_H_10_ were extremely enriched in the initial hydrate phase compared to in the other two systems. A decrease in C_2_H_6_ and iso-C_4_H_10_ concentrations was observed after 4 h with an increase in CH_4_ and C_3_H_8_ in the semi-closed system. This trend was quite similar to that in the open system, but it was much more pronounced in the semi-closed system. For the resulting hydrate phase at a steady state, the composition changed to 54.8 mol% CH_4_, 4.7 mol% C_2_H_6_, 35.5 mol% C_3_H_8_, 3.8 mol% iso-C_4_H_10_, and 1.1 mol% n-C_4_H_10_. This average composition of the hydrate phase at steady state did not yet correspond to the CSMGem calculated composition of the hydrate phase at equilibrium state with 59.8 mol% CH_4_, 2.7 mol% C_2_H_6_, 32.1 mol% C_3_H_8_, 4.8 mol% iso-C_4_H_10_, and 0.7 mol% n-C_4_H_10._ Since the composition of the gas phase in the semi-closed system contained less CH_4_ and more C_3_H_8_ than the gas mixture used in the experiments in the open system, a higher C_3_H_8_ and a lower CH_4_ concentration were detected in the resulting hydrate phase. Apart from C_3_H_8_, iso-C_4_H_10_ and n-C_4_H_10_ were also enriched in the hydrate phase under this circumstance.

## 3. Discussion

Our time-resolved Raman spectroscopic investigations (Figure 2) during the formation process showed a faster incorporation of CH_4_ and C_2_H_6_ into the hydrate phase followed by the enclathration of C_3_H_8_ and iso-C_4_H_10._ This observation of the enclathration sequence was independent of the gas supply conditions. A possible explanation for this phenomenon may be found in the nucleation process. Historically, there are three main hypotheses proposed to explain the hydrate nucleation mechanism. First, Radhakrishnan and Trout [32] proposed the local structuring nucleation hypothesis by studying water–CO_2_ mixtures. They assumed that the guest molecules are arranged in a configuration similar to that in the hydrate crystal due to the thermal fluctuations. Afterwards, the water molecules rearrange around the locally ordered guest molecules to form a hydrate structure. Second, Long [33] and Kvamme [34] presented their model of heterogeneous nucleation at the interface between the gas phase and the liquid phase. Gas molecules are assumed to be absorbed at the aqueous surface where they are at first partially and thereafter completely encased into water cavities. These clusters agglomerate and grow from the water surface to the gas phase. The third hypothesis is that of labile cluster growth, proposed by Sloan et al. [35]. The quintessence of this hypothesis is the formation of labile clusters by water molecules around the dissolved guest molecule. These labile clusters agglomerate by sharing faces until they reach a critical size for hydrate growth. The assumption is also supported by other studies where the existence of water cavities arranged in a pentagonal dodecahedron [36] and ring structures [37] were postulated.

It is experimentally challenging to verify which of these nucleation mechanisms is correct. However, the labile cluster hypothesis shows a higher universal validity compared to the other two hypotheses and may explain the enclathration sequence observed in our experiments.

**Figure 7:**
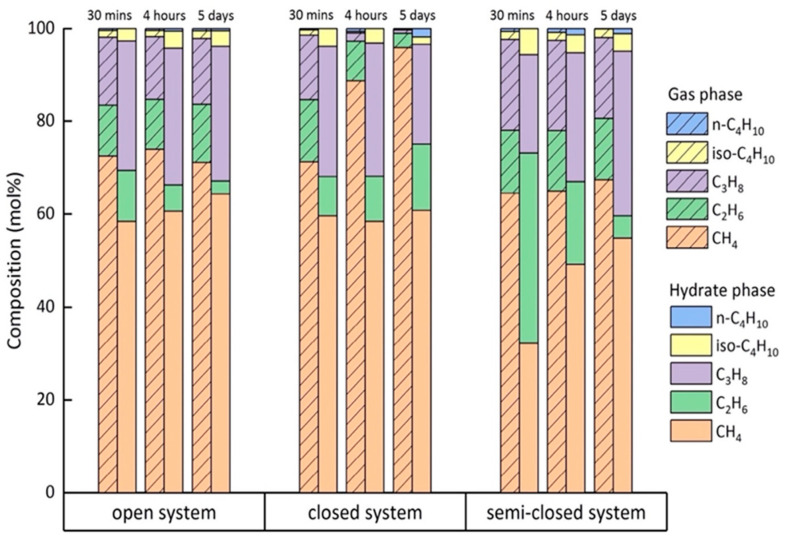
Comparison of actual gas phase composition (shaded slash) with the respective composition in the hydrate phase (blank) in all three systems.

It is widely accepted that driving force for hydrate formation is the difference in chemical potentials between the aqueous phase and the hydrate crystals. To induce hydrate formation, the aqueous phase needs to be supersaturated as the first step [38]. Based on the labile cluster hypothesis, Walsh et al. [39] and Jacobson et al. [40,41] suggested that the dissolved CH_4_ molecule, which is surrounded by 20 water molecules in the aqueous phase, can easily transform into CH_4_ encased in a 5^12^ cavity, whereas the formation of other cavity types is hindered because it requires the incorporation of additional water molecules. This might be a possible explanation for the short induction time of CH_4_, as shown in the Raman spectra of our study. Additionally, the initial concentration of CH_4_ in the gas phase was the highest of all the guest components in this experiment. As for C_2_H_6_ and C_3_H_8_, their solubilities in the water phase may play a significant role. Table 3 tabulates the properties of guest molecules as well as the guest-to-cavity ratios of each component in sII hydrates. Commonly, C_2_H_6_ and C_3_H_8_ have slightly higher solubilities under specific p-T conditions compared to other components. Therefore, a higher concentration of C_2_H_6_ and C_3_H_8_ can be expected in the initial water phase, which certainly leads to faster detection of these two components in the hydrate phase. Interestingly, however, the less soluble iso-C_4_H_10_ is also incorporated into the hydrate structures at the same time as C_3_H_8_. This is probably due to a high guest-to-cavity ratio of iso-C_4_H_10_ in the 5^12^6^4^ cavities of an sII hydrate. A higher guest-to-cavity ratio is probably also the reason for the observation that the concentration of C_2_H_6_ decreased gradually over time, whereas the concentration of C_3_H_8_ and iso-C_4_H_10_ either increased or at least was maintained in the hydrate phase. It is well known that hydrate cavities are stabilized by the inclusion of guest molecules. According to previous research by Lederhos et al. [42], a guest-to-cavity ratio between 0.75 and 1.0 is conducive to stabilizing clathrate cavities. In this case, the ratio for C_2_H_6,_ C_3_H_8_, and iso-C_4_H_10_ were all within this range, according to the results shown in Table 3. Among them, C_3_H_8_ and iso-C_4_H_10_ were expected to best stabilize the large 5^12^6^4^ cavities since the guest-to-cavity ratio was close to 1.0, which indicated a minimum energy. Our observation of the preferred encasement of C_3_H_8_ and iso-C_4_H_10_ in the hydrate phase throughout the whole formation process was in good agreement with the findings from previous studies [22,27]. The situation for n-C_4_H_10_ is much more complicated. It should be noted that n-C_4_H_10_ has two well-known rotational isomers, trans and gauche, since it is a linear alkane and the CH_3_CH_2_ fragments can rotate around the central C–C bond. The trans form is the energetically most stable conformer of n-C_4_H_10_ [43]_,_ but it is supposed to be too large even to fit in the 5^12^6^4^ cavities. Thus, only the gauche conformation of n-C_4_H_10_ can be incorporated into the distorted cage structures [44] and an optimization process of the hydrate structure is requested before the successful incorporation of n-C_4_H_10_. Nevertheless, the detected average composition of the hydrate phase was also in good agreement with the hydrate composition at equilibrium state calculated with CSMGem. Figure 7 compares the actual gas composition with the respective composition of the formed hydrate phase in three separate systems.

In the closed system, the absolute pressure in the sample cell decreased gradually due to the enclathration of guest molecules into the hydrate structures (Figure 3). Nevertheless, the p-T conditions were still within the stability field of a mixed gas hydrate as estimated by the CSMGem. In other words, a coexisting hydrate phase with different composition compared to the initial formed hydrate phase could have formed in the closed system from the changing gas phase. After the first 4 h the measured hydrate crystals showed quite low variations regarding the composition (Figure 4 and Figure S1). We therefore calculated the thermodynamically stable hydrate composition in CSMGem, applying the real-time pressure and gas phase composition after 30 min, 4 h, and 5 days. It is noticeable that the measured composition of the hydrate phase after 30 min corresponded approximately to the equilibrium composition calculated by CSMGem. However, the hydrate composition after 4 h showed a much higher C_3_H_8_ concentration and a lower CH_4_ concentration compared to the predicted equilibrium composition. The hydrate phase after 5 days deviated completely from the calculated results. It was also not possible to detect any coexisting hydrate phase in addition to the original hydrate phase, which had a composition corresponding to the p-T conditions and the composition of the actual gas phase at that time. As indicated in Figure 3, the formation of hydrates took place mainly within the first few hours. Thereafter, the formation process slowed down with the decreasing pressure in the system. In the framework of this experiment (5 days), the system was far from the equilibrium state but slowly adapted to the changes in the gas phase (Figure 7).

For the results detected in the semi-closed system, the composition of the analyzed 12 hydrate crystals was highly heterogeneous and varied strongly within the first 4 h. Unlike the gas phase in the open system with a continuous gas flow, the pressure in the semi-closed system decreased slightly due to the hydrate formation until the pressure regulator was initialed to adjust the pressure in the sample cell again to the original value by pumping in fresh gas. During the time when the pressure was slowly decreasing, individual components could have become depleted in the gas phase. These gas components, possibly due to diffusion processes, may have flow in following the concentration gradient. The frequent turbulence caused by the re-pressurization process in the gas phase may be a potential explanation for the high heterogeneity that occurred within the first 4 h. As the hydrate formation process slowed down after 4 h, the frequency of pressure regulation also decreased, resulting in a lower heterogeneity in the hydrate composition. Nevertheless, the measured composition of the hydrate phase did not reach the calculated composition at equilibrium state (calculated with CSMGem). It should also be mentioned that some of the selected crystals, especially those with smaller sizes in the semi-closed system, disappeared during the ongoing process and therefore it was not possible to continuously characterize the changes throughout the whole experiment. Instead, new crystals were selected for the Raman measurement during the process. This disappearance of smaller crystals is reminiscent of a process similar to Ostwald ripening [45,46].

## 4. Materials and Methods

### 4.1. Experimental Apparatus

The experimental apparatus consisted of a custom-made high-pressure cell made of Hastelloy with a volume of 550 µL in the sample chamber. A pressure regulator (ER3000, Tescom, Emerson, Elk River, MN, USA) adjusted the pressure in the cell, which can be up to 10 MPa with a precision of 2% (relative). A thermostat controlled the temperature of the sample cell by means of a Peltier-cooling device (Type CP1.4-127-045L, Laird Technologies, Santa Clara, CA, USA) with a precision of 0.1 K. The gas flow was measured and regulated with a commercial flowmeter (HI-TEC digital mass flow controller F230M-AAD-11-K, Bronkhorst, Ruurlo, The Netherlands) at a rate of 1 cm^3^/min. To avoid temperature gradients, a supply line with a diameter of max 1.6 mm and a length of 45 mm for the feed gas was drilled into the cell body to cool the incoming gas before entering the sample chamber. Furthermore, a transparent quartz window (diameter 18 mm/thickness 10 mm) equipped on the top of the cell allowed for visual observations of the process in the sample area and the in situ Raman spectroscopic measurements in the forming hydrate phase. By controlling the two valves located at the entry and exit of the pressure cell, the system can be operated with a continuous gas flow, and in other cases, operated with a reduced or interrupted gas flux, simulating the natural reservoir conditions.

The sample cell was integrated into a motorized, software-controlled, Märzhauser Scan+ sample stage attached to the microscope. A confocal Raman spectrometer (LABRAM HR Evolution, Horiba Jobin Yvon, Bensheim, Germany) with 1800 grooves/mm grating and a 20× microscope objective was used for the in situ Raman measurements. The excitation source was a frequency-doubled Nd:YAG solid-state laser with an output power of 100 mW working at 532 nm. With a focal length of 800 mm, this spectrometer achieves a maximum spectral resolution of 0.5 cm^−1^. Specifically, a clarification of the spectral resolution under the chosen experimental conditions was carried out using the radiation spectrum of a neon light. The spectral resolution (full width at half maximum of the 1706 cm^−1^ band for a neon light, Horiba Jobin Yvon, Bensheim, Germany) reached around 0.6 cm^−1^ for this experiment. A motorized pinhole in the analyzing beam path enabled us to variably increase the spatial resolution of laser-spot measurements, which in the x-y direction was 0.5 µm and 1.5 µm in the z-direction. A Silicon Raman band (521 cm^−1^) was employed for the calibration of the Raman band positions before the experiments. More details regarding the pressure cell and experimental setup can be found elsewhere [47].

### 4.2. Experimental Procedures

**Figure 8:**
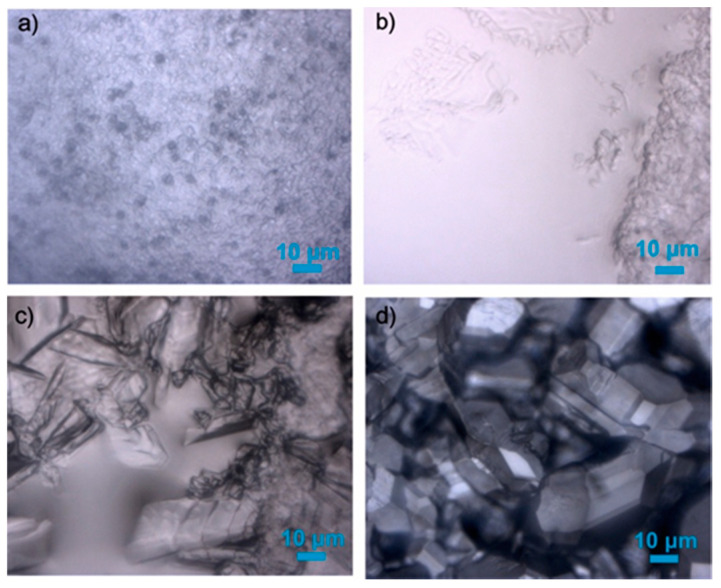
Steps of mixed gas hydrate synthesis from liquid water. (**a**) Formation of ice and mixed gas hydrates, (**b**) melting of ice with only a few hydrate structures left, (**c**) formation of mixed gas hydrates at a temperature above ice point, and (**d**) euhedral hydrate crystals.

Mixed gas hydrates were formed from deionized water and a complex gas mixture supplied as certified gas by Rießner-Gase GmbH. The ordered gas composition was similar to the gas mixtures released from natural hydrates recovered in QMP, containing 71 mol% CH_4_, 11 mol% C_2_H_6_, 15 mol% C_3_H_8_, 2 mol% iso-C_4_H_10_, and 1 mol% n-C_4_H_10_ [11]. Prior to the synthesis of mixed gas hydrates, the pressure cell and supply lines were flushed with the respective gas mixture for about one hour with a gas flow of 1 mL/min. Thereafter, the sample cell was filled with 150 μL deionized and degassed water, carefully sealed, and pressurized with the respective gas mixture. When the final pressure was reached and the flowrate was constant, the cell was cooled down to 253 K to induce the spontaneous crystallization of hydrates and ice. After the formation of hydrates and ice, the cell was slowly warmed up to allow the dissociation of ice and most hydrate crystals until only a few hydrate crystals were left. Subsequently, the cell was cooled down again to a temperature within the stability field of the hydrate phase, but above the melting temperature of the ice. Under these set conditions, euhedral gas hydrate crystals were allowed to grow. This “melting–cooling” process was carried out three times before the p-T condition was fixed at 2.20 MPa and 274 K for the formation of mixed gas hydrates. The moment when the p-T condition first reached 2.20 MPa and 274 K was defined as time zero (*t* = 0 min). Figure 8 shows the pictures of the hydrate formation process that was observed in the pressure cell.

To investigate the hydrate formation process simulating natural reservoir conditions, three different test scenarios were carried out with different gas flows but under identical p-T conditions (Table 4). In test scenario 1, the inlet and outlet valves outside the sample cell were kept open throughout the whole experiment, mimicking a continuous gas supply in natural gas hydrate reservoirs. Test scenario 2 was carried out with the inlet and outlet valves being closed right after initial pressurization to mimic a system with a limited gas supply. To simulate a condition that the hydrate reservoir was partially closed, the outlet valve was closed in test scenario 3 and the inlet valve was open. In this case, the gas flow was regulated in the sample cell, but not continuously, as in the first case of the continuous gas flow. In all these three tests, at least 12 hydrate crystals were selected and continuously characterized with single-point Raman measurements to record the formation process of mixed gas hydrates. Typically, it took 5–6 days until the hydrate reached a steady state where no further changes in the hydrate compositions with regard to the cage occupancy were recorded. Please note that the term “steady state” is used in this context for a state in which the composition of the hydrate phase remains approximately constant for hours. The composition of the hydrate phase at a steady state, however, does not necessarily correspond to the composition of the equilibrium state [48]. Each test was repeated at least two times and similar results were achieved, as discussed in the following sections. All experimental data are available through GFZ Data Services [49].

Since the integrated intensity of the Raman band of each component was proportional to the number of molecules present in the sample, the comparison of the integrated intensities of the Raman bands (band area) could be used for a semi-quantitative determination of the composition of the gas molecules encased in the hydrate crystal at each measuring point [50]. For the determination of the band areas in the Raman spectra, the software package Labspec 6.5 was used. Each spectrum was fitted to a Gauss/Lorentz function after an appropriate background correction to estimate the band areas and positions (Raman shift). The Raman band areas were then corrected with wavelength-independent cross-section factors for each specific component. It was assumed that the cross-section factors did not vary with pressure, inclusion of the component into different cage types of the hydrate phase, or the presence of other components in different phases [50,51,52]. The composition of the guest molecules in the hydrate phase was given as mol%, assuming that the total Raman band areas for the guest molecules were set to 100%. This calculation method is described elsewhere in more detail [53]. Given the fact that we compare and discuss the relative changes of hydrate composition formed under different gas supply conditions, this semi-quantitative analysis of the hydrate composition, which was also successfully applied in our former experiments, was chosen. The more precise method developed by Qin and Kuhs [54] for the determination of the hydrate composition was not considered.

## 5. Conclusions

In this study, we present the results from our experiments simulating the mixed gas hydrate formation in nature by applying in situ Raman spectroscopic measurements and microscopic observations. Based on the background in QMP, the experiments were carried out with a complex gas mixture under three different gas supply conditions, namely, the open, closed, and semi-closed systems. Despite the fact that the initial feed gas was almost the same for three experiments, the composition of the gas phase in the pressure cell changed during the experiment in the different systems and therefore the composition of the resulting hydrate phases differed significantly. The semi-quantitative analysis of both the gas phase and hydrate phase during the formation process provided important information about the gas fractionation and high selectivity of guest molecules encased in hydrate structures with regard to reservoir gas supply conditions. The following points summarize the important findings in this study.

1. The time-resolved characterization of the hydrate phase provides insights into the evolution of guest molecules in the hydrate phase over the formation period. The gas enclathration sequence into the hydrate structures and the varying hydrate composition indicate a complex competition among different gas molecules incorporated into the hydrate crystals depending on their guest-to-cavity ratio and solubility.

2. In the open system with a continuous gas supply, C_3_H_8_ and iso-C_4_H_10_ were enriched in the hydrate phase. A possible reason for this observation is the higher guest-to-cavity ratio of these molecules compared to the other available gas molecules.

3. In the closed system with no gas supply after initial pressurization, a limit of gas supply led to the enclathration of components such as C_2_H_6_ and n-C_4_H_10_, which were not preferentially encased into the hydrate phase in the open system.

4. In the semi-closed system, the changes in hydrate composition over time shared a similar trend with that in the open system; however, this trend was much more pronounced in the semi-closed system. Frequent turbulence in the gas phase caused by pressure regulation between two gas surges may have led to a higher heterogeneity.

5. Despite the fact that the formation of a coexisting hydrate phase would be theoretically possible in the closed system, we did not observe the formation of such an additional coexisting hydrate phase with a composition corresponding to the p-T condition and the composition of the actual gas phase.

## Figures and Tables

**Table 1:** Assignments of measured Raman bands based on literature data.

Component	Vibrational Mode	Cavity Type	ν_measured_ (cm^−1^)	ν_literature_ (cm^−1^)	References
CH_4_	C–H symmetric stretching	sII 5^12^	2912	2912	[27]
		sII 5^12^6^4^	2901	2901	
C_2_H_6_	C–C symmetric stretching	sII 5^12^6^4^	991	991	[10]
C_3_H_8_	C–C symmetric stretching	sII 5^12^6^4^	876	876	[10]
iso-C_4_H_10_	C−C symmetric stretching	sII 5^12^6^4^	811	811	[27]
n-C_4_H_10_	C–C symmetric stretching	sII 5^12^6^4^	838 (gauche form)	838	[26]

**Table 2:** Average composition of the hydrate phase formed under different gas supply conditions.

Experimental Condition	Time	Average Concentration of Each Component in the Hydrate Phase (mol%)				
		CH_4_	C_2_H_6_	C_3_H_8_	iso-C_4_H_10_	n-C_4_H_10_^1^
Open	After 30 min	58.4	11.1	27.8	2.7	0.0
	After 4 h	60.6	5.7	29.5	3.7	0.6
	After 5 days	64.3	2.8	29.0	3.3	0.5
Closed	After 30 min	59.6	8.5	28.0	3.9	0.0
	After 4 h	58.4	9.8	28.7	3.1	0.0
	After 5 days	60.8	14.3	21.5	1.6	1.8
Semi-closed	After 30 min	32.2	41.0	21.2	5.6	0.0
	After 4 h	49.1	17.9	27.8	3.9	1.3
	After 5 days	54.8	4.7	35.5	3.8	1.1

^1^ n-C_4_H_10_ was not detected in the Raman spectra after 30 min.

**Table 3:** Properties of guest molecules as well as the guest-to-cavity ratio of each component in the sII hydrate. Except for the solubility data from Air Liquide Germany GmbH, all the other data were from Sloan and Koh, 2008 [2].

Molecule	Structure	Solubility (mmol/L) ^1^	Guest Diameter (Å)	Guest-to-Cavity Ratio	
				5^12^ (sII)	5^12^6^4^ (sII)
CH_4_	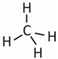	1.6	4.36	0.87	0.66
C_2_H_6_	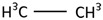	2.2	5.50	1.10	0.83
C_3_H_8_	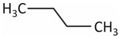	1.8	6.28	1.25	0.94
iso-C_4_H_10_	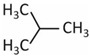	1.0	6.50	1.29	0.98
n-C_4_H_10_	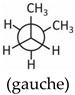	1.6	7.10	1.41	1.07
	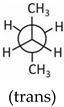		7.86	1.57	1.18

^1^ Solubility in water at 20 °C and under normal pressure.

**Table 4:** Experimental conditions of the 3 parallel tests regarding the inlet valve and outlet valve of the Raman pressure cell.

	Gas Inlet	Gas Outlet	System
**Test Scenario 1**	Open	Open	Open system
**Test Scenario 2**	Closed	Closed	Closed system
**Test Scenario 3**	Open	Closed	Semi-closed system

## Data Availability

Research data associated with this article can be accessed through GFZ Data Services at https://doi.org/10.5880/GFZ.3.1.2021.003, accessed on 24 May 2021. It should be cited as: Pan, Mengdi; Schicks, Judith M. (2021), Raman spectroscopic data from gas hydrates formed from a complex gas mixture with different gas supply conditions. GFZ Data Services.

## Supplementary Materials

The following is available online, Figure S1. Composition changes measured on the surface of mixed hydrate crystals during a repeated test in the open system (Figure S1a), the closed system (Figure S1b), and the semi-closed system (Figure S1c).

## Sample Availability

Samples of the compounds are not available from the authors.
