# Supplementary material for: Influence of Gas Supply Changes on the Formation Process of Complex Mixed Gas Hydrates
Source: Molecules. 2021 May 19;26(10):3039. doi: 10.3390/molecules26103039 (PMC8160831; doi:10.3390/molecules26103039)
Supplement: Supplementary file 1 [file molecules-26-03039-s001.zip › molecules-1217621-supplementary.pdf]

Supporting Information

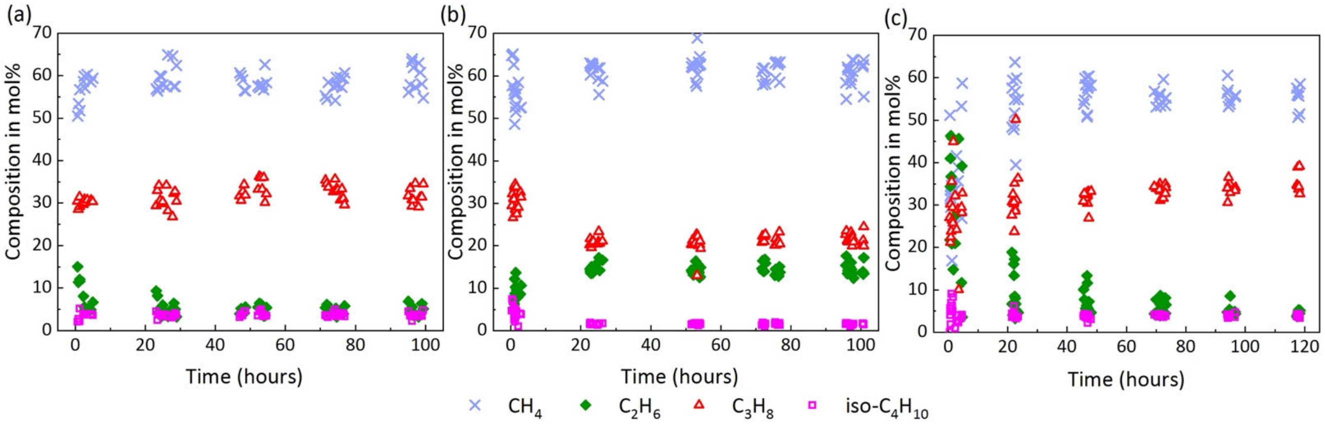

Figure S1. Composition changes measured on the surface of mixed hydrate crystals during a repeated test in the open system (Figure S1a), the closed system (Figure S1b), and the semi-closed system (Figure S1c).
